# Evaluation of research topic evolution in psychiatry using co-word analysis

**DOI:** 10.1097/MD.0000000000007349

**Published:** 2017-06-23

**Authors:** Ying Wu, Xing Jin, Yunzhen Xue

**Affiliations:** a School of Humanities and Social Sciences; b Affiliated Tumor Hospital, Shanxi Medical University, Taiyuan, China.

**Keywords:** co-word analysis, psychiatry, topic evolution

## Abstract

With the rapid increase in the incidence of mental disorders and mental issues, psychiatry has become one of the fastest growing clinical medical disciplines. Development priorities and research foci in this field have evolved over different periods.

All the articles in 10 psychiatric journals with the highest impact factors were selected from the Science Citation Index (SCI) in Web of Science from 2001 to 2015. The information visualization software Sci^2^ was used to conduct co-word and clustering analyses on these articles. The articles were divided into 3 periods: 2001 to 2005, 2006 to 2010, and 2011 to 2015. Each bibliographic record contained a title, author names, abstract, keywords, references, and other information.

During the 3 periods between 2001 and 2015, child and adolescent psychiatry, major depression, schizophrenia, and prefrontal cortex were constant research foci. The brain and meta-analysis gradually became new research foci, although research on symptoms slowly decreased. Molecular genetics was also an area of interest.

Using scientometrics technology to visualize research foci can provide us with new ideas and research methods. Co-word analysis for the preliminary exploration of research foci and developmental trends in psychiatry is helpful in finding developmental rules, choices of topics, and innovative research. Our study had some limitations. In the future, we should expand our research scope and use a variety of research methods to enrich our results.

## 1 Introduction

Mental disorders are harmful to human health and seriously affect social life. There are already more than 4.5 billion patients with mental disease in the world.^[1]^ According to a recent survey of the World Health Organization, it has been estimated that mental disorders rank the first in terms of disability-adjusted life years (DALYs), surpassing cardiovascular disease, respiratory system disease, and malignant tumor.^[2–4]^ Due to the rapid increase in cases of mental disorder and mental issues, psychiatry has become one of the fastest growing medical disciplines. In recent years, with new techniques and concepts coming, psychiatry is rapidly crossing and fusing with subjects such as genetics, imaging, and psychology. Research on hotspots and developmental trends of the discipline has important significance not only for researchers but also clinicians. Keywords represent the key points of the literature and the research foci of many scholars.^[5]^ By analyzing the frequencies of keywords and clustering those keywords, we can understand the development priorities and research foci in different periods. In this study, the visualization instrument Sci^2^ was used to conduct co-word analysis of keywords. The co-word analysis method was first proposed by Callon et al.^[6]^ This is an important bibliometric method that includes citation analysis, coauthor analysis, and co-word analysis. Co-word analysis can be used to investigate research topics or the direction of research in a discipline. This method uses professional terms that appear in the same paper to judge the relationships among subjects and research structures to find current foci of research in the field. Due to the wide range of current subjects of the study, and the scattered and unstable nature of cited reports, the use of keywords can be used to study emerging areas of research reflecting the development of a field of study.^[7]^ More frequently used keywords normally emerge more frequently in the co-occurrence matrix and have larger the nodes on the map. In this map, Sci^2^ can not only reveal the keyword hierarchy, but can also clearly highlight key areas of development during different periods by monitoring changes in the nodes.^[8]^ Sci^2^, with its advantage in visualization, was developed by Katy Borner and her team at Indiana University.^[9]^ It has unique functional advantages in the construction of maps. It not only supports input of data with a variety of formats, but also contains strong tools for data analysis. It can be used to automatically construct different networks using many kinds of algorithms, such as co-word analysis networks and literature citation coupling networks. Sci^2^ also has powerful function of extending. The software now has integrated visual tools, such as GUESS, Cytoscape, and Gephi.R. In addition, researchers can further expand the software according to their needs over time. When this software is used for co-word clustering, papers can be categorized using themes and divided into several categories. We used scientometrics technology to visually research areas of focus and new ideas and research methods. Co-word analysis was used for the preliminary exploration of research focus and developmental trends in the field of psychiatry. Our research may be helpful in revealing developmental rules, new topics of study, and innovative research.

## 2 Methods

We selected 54,647 documents from the top 10 psychiatric journals according to the JCR (Journal Citation Reports) in 2014. Articles with the highest impact factors, as indicated in the Science Citation Index (SCI) were selected in Web of Science from 2001 to 2015 (Table 1). Each bibliographic record contained the title, author names, abstract, keywords, references, and other information. These 15 years were then divided into 3 periods of time: from 2001 to 2005, from 2006 to 2010, and from 2011 to 2015. Then the domains used to map knowledge were constructed and the articles from the 3 periods were analyzed.

**Table 1:**
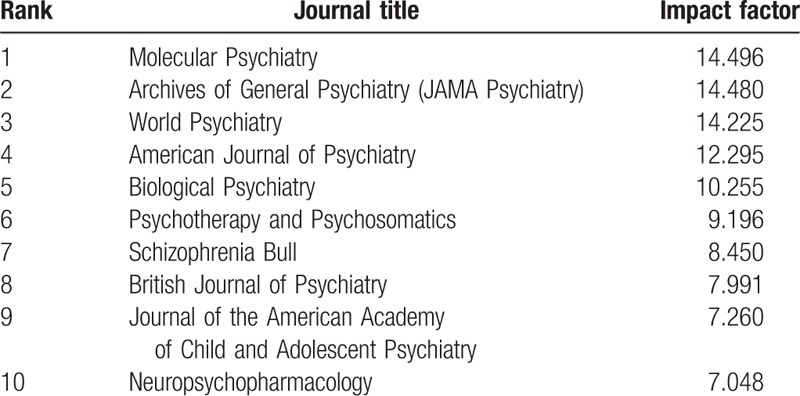
Ten representative journals in the field of psychiatry.

For co-word analysis using Sci^2^, the keywords were first standardized and then used to build a co-occurrence network. After analyzing the nodes and lines in the network, the isolated nodes were deleted. We used the minimum spanning tree (MST)-Pathfinder algorithm to reduce the number of lines and highlight the most important lines. This led to the construction of visual networks of the field of psychiatry from 2001 to 2015 (Table 2). By examining the frequencies of other keywords and the distances from these keywords to the core keyword, the topics under discussion were determined in each subnetwork.

**Table 2:**
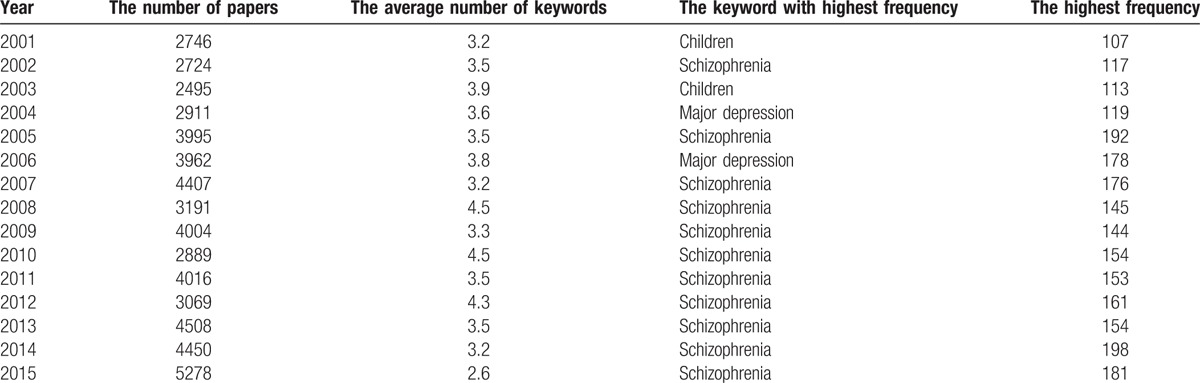
The frequencies of keywords from 2001 to 2015.

Ethical approval was not necessary for this study, because all the data were obtained from Web of Science on the Internet.

## 3 Results

### 3.1 Co-word network from 2001 to 2005

**Figure 1:**
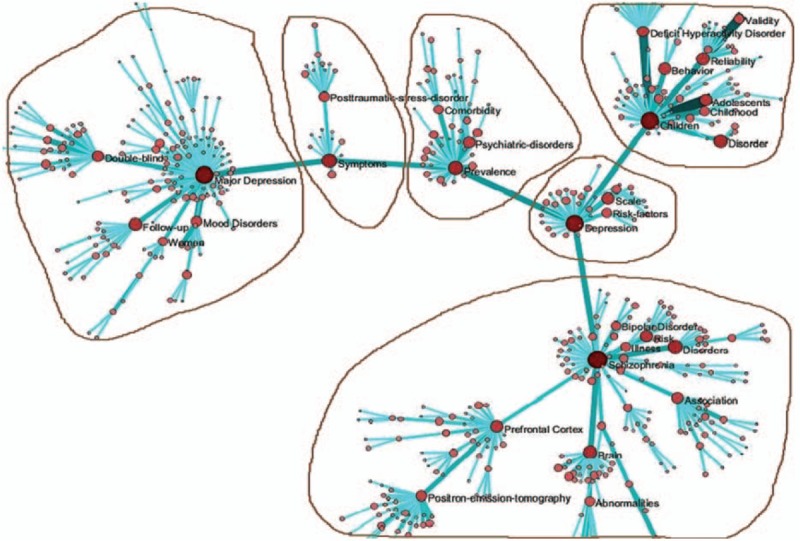
Co-word analysis map from 2001 to 2005.

From 2001 to 2005, there were 14,871 records. The keywords were first standardized and the co-word networks were selected. There were 25,234 nodes, among which 14,791 were isolated nodes. We obtained co-word networks with 10,443 nodes and 154,625 lines. To highlight the co-words and to reduce the scale of the network, the edge weight was set to >5 and the MST-Pathfinder algorithm was applied to retain 646 nodes and 632 sides. We accepted 6 core keywords embraced by 6 subnetworks and selected high-frequency keywords more than 150 times (Table 3 and Fig. 1).

**Table 3:**
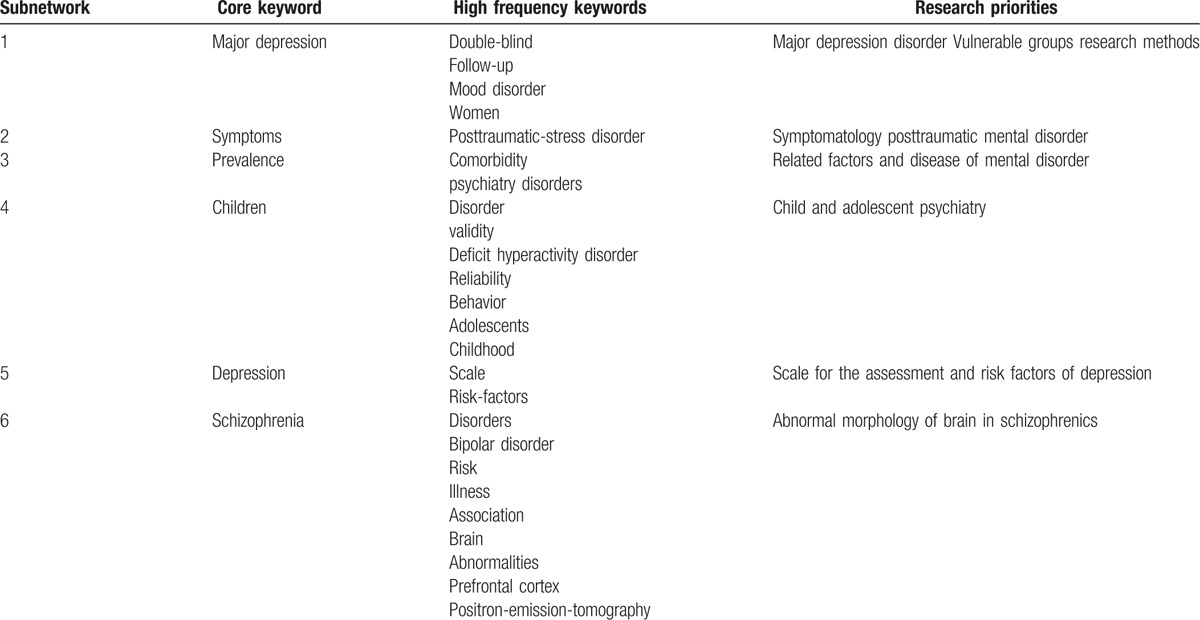
Keywords from articles written between 2001 and 2005.

The core keyword in the first subnetwork was major depression, with a frequency of occurrence (FO) of 543. Thus, major depression disorder (MDD) was the central research focus in this subnetwork. By examining the frequency of other keywords and the distance from these keywords to the core keyword, this subnetwork mainly discussed vulnerable groups and research methods for MDD.

The core keyword in the second subnetwork was symptoms, with a frequency of occurrence of 354; symptomatology was the central research focus in this subnetwork. This subnetwork mainly discussed post-traumatic stress disorder.

The core keyword in the third subnetwork was prevalence, with a frequency of occurrence of 343. The related factors of mental disorders were the central research focus in this subnetwork. This subnetwork mainly discussed disorders comorbid with mental disorders.

The core keyword in the fourth subnetwork was children (FO  =  524). Child and adolescent psychiatry was the central research focus in this subnetwork. This subnetwork mainly discussed the diagnostic criteria of attention-deficit hyperactivity disorder.

The core keyword in the fifth subnetwork was depression, with an FO of 498. Depression was the central research focus in this subnetwork. This subnetwork mainly discussed scales for the assessment of and risk factors for depression.

The core keyword in the sixth sub-network was schizophrenia (FO  =  601). Schizophrenia was the central research focus in this subnetwork. This subnetwork mainly discussed abnormal morphology of the brain in schizophrenia.

### 3.2 Co-word network from 2006 to 2010

**Figure 2:**
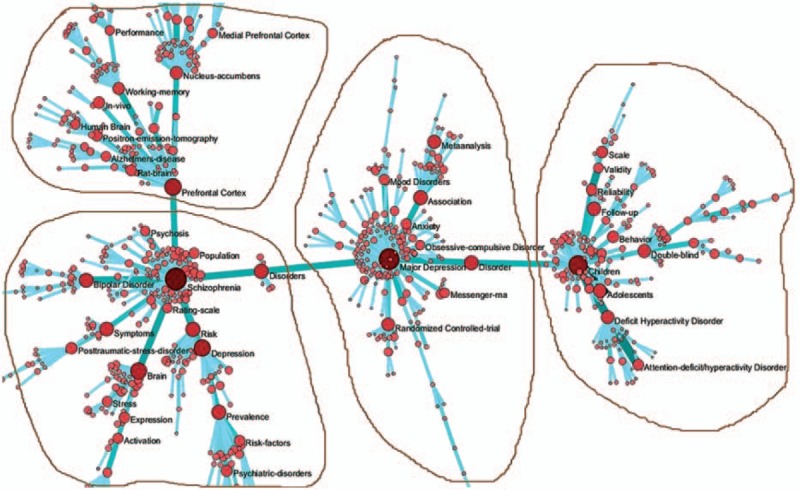
Co-word analysis map from 2006 to 2010.

We found 18,453 records from the time period between 2006 and 2010. When the keywords were standardized and the co-word networks were selected, there were 32,418 nodes, among which 18,381 nodes were isolated. We obtained a co-word network with 14,307 nodes and 220,687 lines. To highlight co-words and reduce the scale of the network, the edge was set to >5 and the Mst-Pathfinder algorithm was applied to retain 844 nodes and 838 sides. We accepted 4 keywords embraced by 4 subnetworks and selected high-frequency keywords more than 150 times (Table 4 and Fig. 2).

**Table 4:**
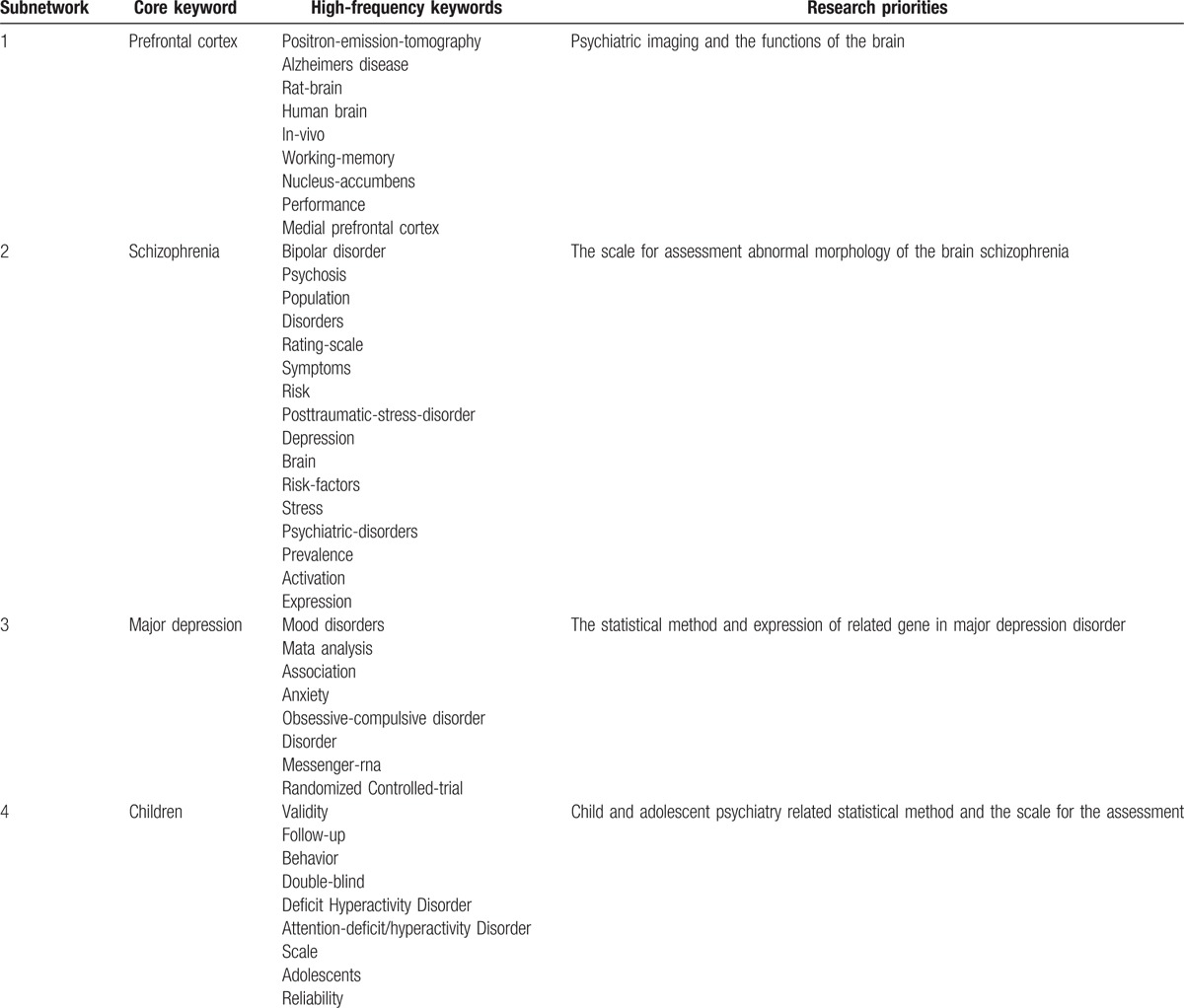
Keywords from articles written between 2006 and 2010.

The core keyword in the first subnetwork was prefrontal cortex (FO  =  454). The prefrontal cortex of the brain was the central research focus in this subnetwork, which mainly discussed psychiatric imaging and the functions of the parts of the brain by examining the frequency of other keywords and the distance from these keywords to the core keyword.

The core keyword in the second subnetwork was schizophrenia, with a frequency of occurrence of 771. Schizophrenia was the central research focus in this subnetwork. By examining the frequency of other keywords and their distances to the core keyword, this subnetwork mainly discussed psychiatric symptoms, scales for the assessment of schizophrenia, and abnormal morphology of the brain in patients with mental disorders.

The core keyword in the third subnetwork was major depression (FO  =  690), with major depression disorder as the central research focus in this subnetwork. This subnetwork mainly discussed statistical methods and the expression of related genes in depression.

The fourth subnetwork had a core keyword of children, with a frequency of occurrence of 591. Child and adolescent psychiatry was also the central research focus in this subnetwork. This subnetwork mainly discussed attention-deficit hyperactivity disorder, related statistical methods, and scales for the assessment of these disorders.

### 3.3 Co-word network from 2011 to 2015

**Figure 3:**
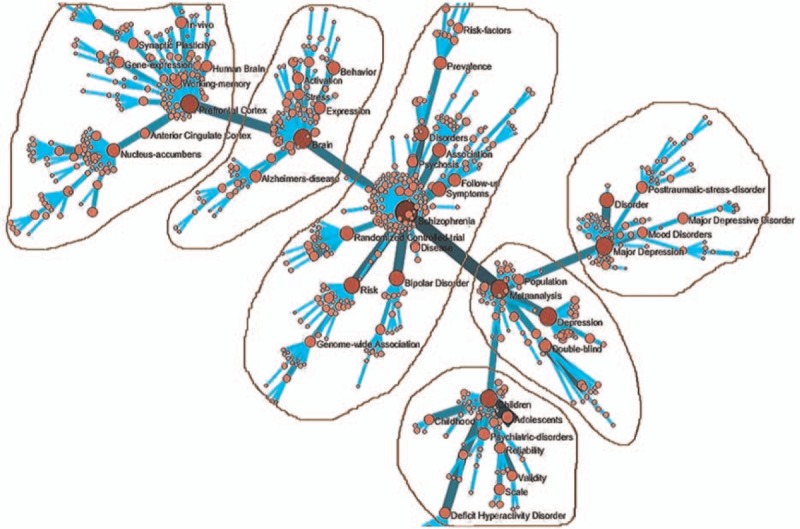
Co-word analysis map from 2011 to 2015.

There were 21,323 records with dates between 2011 and 2015. After the keywords were standardized and co-word networks were selected, there were 34,939 nodes, among which 21,142 nodes were isolated. We obtained a co-word network with 13,797 nodes and 215,523 lines. To highlight co-words and to reduce the scale of the network, the edge weight was set to >5 and the MST-Pathfinder algorithm was applied to retain 799 nodes and 784 sides. We accepted 6 keywords embraced by 6 subnetworks and selected high-frequency keywords more than 150 times (Table 5 and Fig. 3).

**Table 5:**
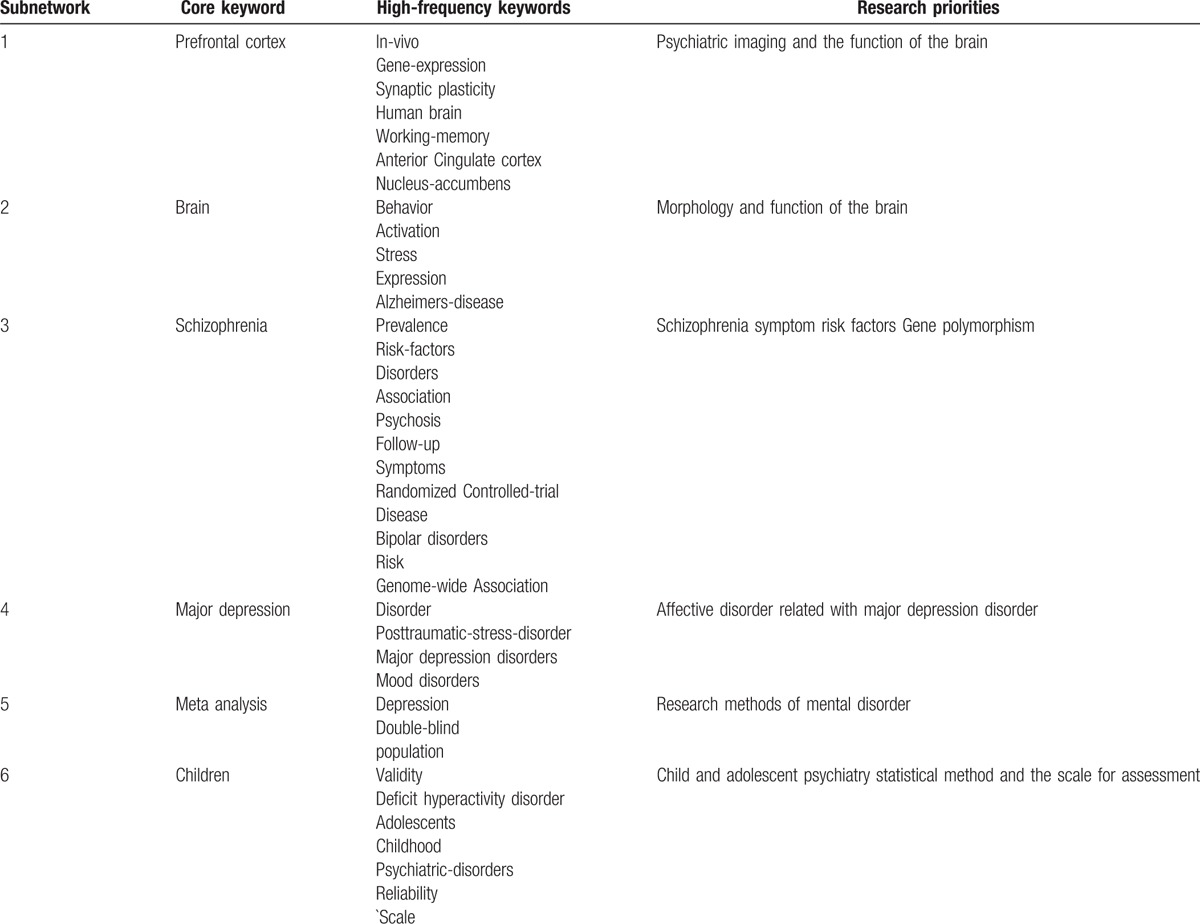
Keywords from articles written between 2011 and 2015.

Again, the core keyword in the first subnetwork was prefrontal cortex (FO  =  454). The prefrontal cortex of the brain was the central research focus in this subnetwork, which mainly discussed psychiatric imaging and the functions of each part of the brain by examining the frequency of other keywords and the distance from these keywords to the core keyword.

The core keyword in the second subnetwork was brain, with a frequency of occurrence of 551. Thus, the prefrontal cortex of the brain was center research focus in this subnetwork. This sub-network mainly discussed the morphology and function of the whole brain in patients with mental disorders.

The core keyword in the third subnetwork was also schizophrenia, with a frequency of occurrence of 847. This subnetwork mainly discussed symptoms, risk factors, and gene polymorphisms of schizophrenia.

The core keyword in the fourth subnetwork was also major depression; in this instance, the frequency of occurrence was 483. This subnetwork mainly discussed affective disorders related to major depression disorder.

The core keyword in the fifth subnetwork was meta-analysis (FO  =  847). This subnetwork mainly discussed research methods regarding mental disorders.

The core keyword in the sixth subnetwork was children, with a frequency of occurrence of 478. Child and adolescent psychiatry was the central research focus in this subnetwork, and this subnetwork mainly discussed attention-deficit hyperactivity disorder, related statistical methods, and scales for assessment, as determined by examining the frequency of other keywords and the distance from these keywords to the core keyword.

## 4 Discussion

Across our co-word analysis of keywords in psychiatry from 2001 to 2015, several factors appeared and changed. In the first stage (2001–2005), the symptoms of mental disorders were a main research direction, focusing particularly on depression and the risk factors and assessment of schizophrenia. In the second stage (2006–2010), the number of subnetworks decreased and was more concise, but the number of keywords with frequencies more than 150 increased and the research themes were more diversified, including the brain and mRNA. Although the brain was not a core keyword, it gradually became an important new research direction. The new keyword mRNA also had a frequency of more than 150, showing that the relationship between molecular genetics and mental disorders was a research hot spot.

In the third stage (2011–2015), there were 2 new networks; one was a subnetwork with brain as its keyword. In this subnetwork, the high-frequency keyword was Alzheimer disease, which is a primary degenerative disease in the central nervous system with diffuse atrophy of the brain as the main pathological change. Accordingly, the changes of brain morphology in mental disorders have received more and more attention from researchers. Another network centered around meta-analysis, a new method of examining mental disorders that has become an important research direction.

Molecular genetics was also a hot topic in psychiatry. Many studies have shown that mental disorders are complex polygenic diseases, so susceptibility genes for mental disorders have been a focus of research. With the great progress in theory and technology in molecular biology, genetic research is developing rapidly. As genome-wide association analysis matures, an increasing number of candidate genes can be found for mental disorders. As this kind of disorder is a heavy burden on society, locating genes corresponding to specific clinical symptoms of mental disorders and designing drug treatment regimens according to relevant genotypes personalized for patients with different mental disorders are significant objectives to be achieved in the treatment of mental disorders.^[10,11]^

In all 3 stages, child and adolescent psychiatry, depression, schizophrenia, and the prefrontal cortex were significant. Meta-analysis gradually became a new research focus in the third stage, but the heat of symptomatology continued to decrease. Therefore, although the traditional research foci cannot disappear suddenly, new research foci will be more diversified in the future. The brain, meta-analysis, and genetic research may be worth more attention.

In the 21st century, psychiatry has developed rapidly. Because of rapid development in numerous basic subjects and wide use of new brain imaging techniques, the application of theory in psychology and sociology attached importance to fundamental changes that occurred in understanding mental disorders to form the biopsychosocial medical model. At present, people can not only delve deep into molecular, but also pay more attention to the pathogenic role of psychosocial factors in mental disorders. The biopsychosocial model, the high level of modern medical theory, and new technical applications for the prevention, diagnosis, and treatment of mental disorders will lead to splendid achievements in psychiatry.

Using scientometrics technology to visualize research foci can provide new ideas and research methods. Using co-word analysis in the preliminary exploration of research foci and developmental trends in psychiatry is helpful in revealing developmental rules, choices of topics, and innovative research.

Our study had some limitations. A single database, Web of Science, was used because this database is more authoritative and analyzed more easily than other databases, such as Medline or Pubmed. In this database, the top 10 magazines were selected and 15 years were divided into 3 stages averagely to reduce computation and find out the key clustering and changing characteristics of disciplinary research hotspots. If the data during 15 years were analyzed totally, it was possible that the number of clustering subnetworks was more numerous not to be confused the primary with secondary and it was difficult to observe the changing characteristics of scientific research hotspots and influence analyzing and judging it. If the amount of data was small such as during 3 years, it may be difficult to observe the change of scientific research hotspots and analyze the result of scientific research. Many software programs are available for co-word analysis. These include UCINET, Pajek, and Vosviewer. We used Sci^2^, which has considerable advantages when analyzing the database in Web of Science, the construction of complex networks, and drawing. This software with the co-word algorithm plug-in unit and drawing plug-in unit has enormous advantages in highlighting important clustering and reducing the edges reasonably. If other software was used, it is possible that the cluster scope was not clear and the map was not simple enough. Future research will encompass the use of different analysis software programs. We will compare these programs to completely evaluate subject hotspots. In the future, we should also expand the research scope and use a variety of research methods to enrich our research results.

## Acknowledgments

The authors thank the participants for their time and willingness to participate in this study. The authors also acknowledge support for this study from the grant ”Analysis on Scientific Collaboration and Trends Prediction of Research Fronts in Psychiatry Field (No. 71503152)” awarded by the National Natural Science Foundation of China. This study was also supported by Program for the Outstanding Innovative Teams of Higher Learning Institutions of Shanxi (OIT).
